# Research Trend of Publications Concerning Antibody-Drug Conjugate in Solid Cancer: A Bibliometric Study

**DOI:** 10.3389/fphar.2022.921385

**Published:** 2022-06-20

**Authors:** Xiangjun Qi, Yanlong Li, Wei Liu, Yifan Wang, Zhuangzhong Chen, Lizhu Lin

**Affiliations:** ^1^ The First Clinical School of Guangzhou University of Chinese Medicine, Guangzhou, China; ^2^ The First Affiliated Hospital of Guangzhou University of Chinese Medicine, Guangzhou, China; ^3^ School of Chinese Classics Guangzhou University of Chinese Medicine, Guangzhou, China

**Keywords:** antibody-drug conjugate, solid cancer, bibliometric analysis, citespace, VOSviewer, histcite, bibliometrix

## Abstract

**Background:** Antibody-drug conjugate (ADC) is a promising therapy for solid cancer that has raised global concern. Although several papers have reviewed the current state of ADCs in different solid cancers, a quantitative analysis of the publications in this field is scarce.

**Methods:** Publications related to ADC in the field of solid cancer were obtained from the Web of Science Core Collection. Data analyses were performed with VOSviewer 1.6.9, HistCite 2.1, CiteSpace V and *R* package Bibliometrix.

**Results:** A total of 3,482 records were obtained in the holistic field and 1,197 in the clinical field. Steady growth in the number of publications was observed. The United States was the leading contributor in this field. Krop IE was the most influential author. The most productive institution was Genentech Inc., while Mem Sloan Kettering Canc Ctr was the most cited one. The most impactful journal was the *Journal of Clinical Oncology*. A total of 37 burst references and five burst references were identified between 2017–2022 in the holistic and clinical fields, respectively. Keywords analysis indicated that ADCs research mainly involved breast cancer, triple-negative breast cancer, ovarian cancer, small cell lung cancer, prostate cancer, gastric cancer, and urothelial carcinoma. ADC agents including trastuzumab emtansine, trastuzumab deruxtecan, sacituzumab govitecan, enfortumab vedotin, and rovalpituzumab tesirine were highly studied. Targets including HER2, trophoblast cell-surface antigen, mesothelin, delta-like ligand 3, and nectin-4 were the major concerns.

**Conclusion:** This study analyzed publications concerning ADCs in the field of solid cancer with bibliometric analysis. Further clinical trials of ADCs and designs of the next generation of ADCs are the current focuses of the field. Acquired resistance of ADCs and biomarkers for ADC therapy efficacy monitoring are future concerns.

## 1 Introduction

The continuous discovery of cytotoxic chemicals from the mid-20th century onward has facilitated the emergence of chemotherapy as the primary antitumor pharmacotherapy (Devita and Chu, 2008). However, its cytotoxicity can also damage normal cells due to a deficiency of specific targets and a precise drug delivery system. The appearance of targeted therapy compensated for the flaws of chemotherapy, and advantages such as the monoclonal antibody technique, identification of novel tumor markers, and antigens promoted the development of more targeted antitumor therapeutics (Liu, 2014). Nanotechnology and nanotherapeutics contribute to the combination of cytotoxic chemicals and antibodies, which are known as antibody-drug conjugates (ADCs).

An ADC consists of a monoclonal antibody (mAb) coupled to a cytotoxic compound (payload) *via* a linker (Chari, 2008). This unique structure of ADCs makes it possible for cytotoxic weapons to efficiently target tumor cells. The first ADC drug was approved for acute leukemia back in 2000 (Norsworthy et al., 2018). However, it was not until 2013 that ADC drugs achieved a breakthrough in the field of solid tumors. Ado-trastuzumab emtansine (T-DM1), which contains a monoclonal antibody targeting human epidermal growth factor receptor 2 (HER2) linked to a payload of microtubule inhibitor DM1 through a non-cleavable thioether linker, was approved for the treatment of HER2-positive breast cancer in 2013 (Boyraz et al., 2013). This has prompted unprecedented enthusiasm for developing ADC drugs as a transformative therapy for solid cancer. There is an increasing number of clinical or preclinical research on ADCs in the field of solid cancer with a rough estimate more than 30 ADCs, 15ADCs, 10 ADCs, 10 ADCs, and 5 ADCs in gastrointestinal malignancies, gynecological malignancies, lung cancer, HER2-positive breast cancer and hepatocellular carcinoma, respectively (Ferraro et al., 2021; Martín-Sabroso et al., 2021; Ricciuti et al., 2021; Singh et al., 2021; Murali et al., 2022). Although several reviews have summarized the current state of ADCs in different solid cancers (Lambert and Morris, 2017; Nagayama et al., 2017; Deonarain and Yahioglu, 2021), a quantitative analysis of publications in this field is scarce.

bibliometric analysis is a method that provides statistical analysis and quantitative to academic publications. Through bibliometric analysis, it is able to draw network knowledge maps, predict new trends and demonstrate the latest developments in a particular field (Guler et al., 2016). Currently, bibliometric analysis has been used in exploring the research trends of cancer drug therapy, such as immune checkpoint inhibitors (Gao et al., 2019). However, bibliometric studies concerning ADCs in solid cancer remain absent.

In the current study, a comprehensive bibliometric analysis was conducted to reveal the research status, research focus, and research trends of ADCs in the field of solid cancer.

## 2. Materials and Methods

### 2.1 Data Source and Collection

A comprehensive literature search was performed on the Web of Science Core Collection (WoSCC) database. The following search terms were combined to filter publications that were related to ADC and solid cancer (antibody-drug conjugate AND cancer) NOT hematologic cancer. We further combined it with “trial” and “meta-analysis” to obtain clinical publications. The final retrieval strategy is presented in Supplementary Table S1. The publication type was restricted to article and review. There was no limitation on the publication date, while the final retrieval was conducted in January 2022. Two researchers conducted the retrieval independently. Disagreements during the retrieval process were discussed with a third colleague or the entire academic team to achieve consensus.

### 2.2 Data Analysis and Tools

HistCite 2.1 software (New York, United States) was used to calculate the publications and citations of countries, institutions, authors, journals, targets, payloads and linkers related to ADC. The total local citation score (TLCS) is the number of citations to the author/journal/reference from papers within our data collection. Elements with a high TLCS are of significance to a given field. VOSviewer 1.6.9 software (Leiden University, Leiden, Netherlands) was used to depict network maps of journals, institutions, and countries and conducted a cluster analysis of high-frequency keywords. In the network maps, different nodes represent elements such as journals, institutions, countries, or keywords, while the size of nodes indicates the number of publications or the frequency of citation. Nodes in different colors represent different clusters or years and links between nodes reflect relationships such as collaboration or citation. The full counting method of VOSviewer and LinLog/modularity method was applied to analyze the network maps.

CiteSpace V is a full-featured bibliometric software designed by Chaomei Chen. CiteSpace V is characterized by revealing dynamics and hotspots in a given field through its function of burst analysis, which can detect topics that change dramatically over a while. Thus, a burst analysis for cited references was conducted to demonstrate the high influential references in the current field. Furthermore, a dual-map overlay of journals was used to analyze the scientific distribution and disciplinary evolution.

The *R* package Bibliometrix (Aria and Cuccurullo, 2017) was used for constructing a collaboration world map to reflect the geographical distribution of publications.

## 3. Results

### 3.1 Publication Language

In the holistic field, the 3,482 records retrieved were published in nine languages. Of the 3,482 records, 3,434 (98.62%) were published in English, 21 (0.60%) in French, 11 (0.32%) in German, 8 (0.23%) in Japanese, 2 (0.06%) in Chinese, Polish, Spanish, and 1 (0.03%) in Portuguese and Russian. In the clinical field, 1,197 records were retrieved and published in six languages. Of the 1,197 records, 1,174 (98.1%) were published in English, 13 (1.1%) in French, 6 (0.5%) in German, 2 (0.2%) in Japanese, and 1 (0.1%) in Portuguese and Spanish.

### 3.2 Publication Outputs

**FIGURE 1:**
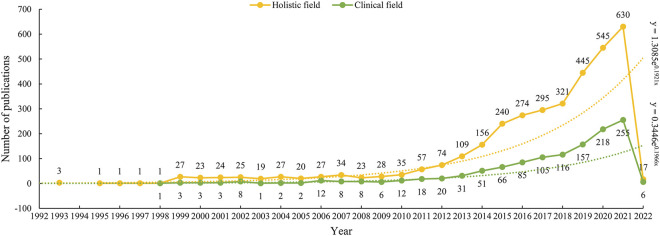
Annual publications of ADC research in the field of solid cancer.

As we can see from Figure 1, the first three studies on ADC research in the field of solid cancer were all published in 1993. There was a silent stage where the annual publication stayed between 0–1 from 1994 to 1998 in the wake of the initial three studies. Between 1999 and 2009, it went into an exploratory stage, and the number of annual publications increased slightly. The number of annual publications started increasing rapidly in 2009. Especially after 2018, the number of annual publications gradually increased from 35 in 2010, 109 in 2013, 240 in 2015, 321 in 2018, to 630 in 2021. From 2018 to 2021, a total of 1941 papers were published, accounting for 55.74% of all the included studies. As for the outputs of clinical research, the publishing trend is similar to that of the holistic ADC field. The first paper on ADC clinical research was published in 1998, and its climax of publication was reached in 2021 with 255 papers coming out.

### 3.3 Countries and Institutions

**FIGURE 2:**
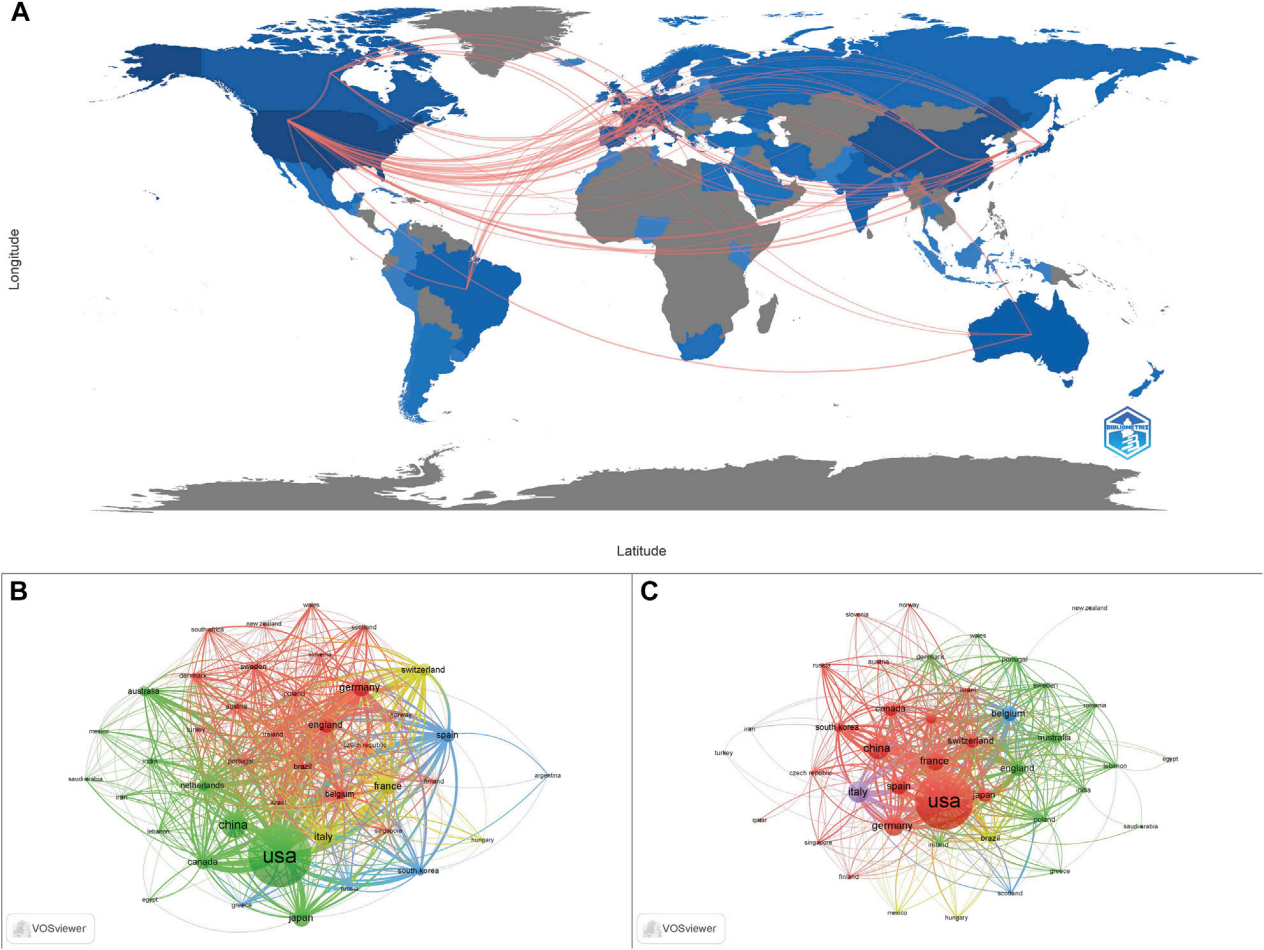
The geographical distribution of the publications and the country collaboration network map. **(A)** The geographical distribution of publications related to ADC research. **(B)** Country collaboration in the holistic ADC research field. **(C)** Country collaboration in the clinical ADC research field.

A total of 75 countries have contributed to the publication of ADC research from 1993 to 2022. As can be seen from the data in Table 1, the top seven countries were the United States (1756, 50.43%), China (455, 13.07%), Germany (270, 7.75%), Italy (265, 7.61%), Japan (253, 7.27%), UK (240, 6.89%), France (233, 6.69%), while the other countries published less than 200 papers. The geographical distribution map is presented in Figure 2A, in which we can see a dense collaboration between the United States and Europe. A collaborative network map of the countries was also plotted. As shown in Figures 2B,C, the United States dominated country cooperation. As for clinical research, the top five countries with publications greater than 100 were the United States (636, 53.13%), Italy (135, 11.28%), China (150, 12.53%), Germany (118, 9.86%), and France (116, 9.69%).

**TABLE 1:** The top 10 countries and institutions contributing to publications of ADC research in the solid cancer field [n (%)].

Holistic Field				
Rank	Country	N (%)	Institution	N (%)
1	United States	1756 (50.43%)	Genentech Inc.	169 (4.85%)
2	China	455 (13.07%)	Mem Sloan Kettering Canc Ctr	130 (3.73%)
3	Germany	270 (7.75%)	Dana Farber Canc Inst	98 (2.81%)
4	Italy	265 (7.61%)	Univ Texas MD Anderson Canc Ctr	98 (2.81%)
5	Japan	253 (7.27%)	NCI	97 (2.79%)
6	United Kingdom	240 (6.89%)	Harvard Med Sch	82 (2.35%)
7	France	233 (6.69%)	ImmunoGen Inc.	63 (1.81%)
8	Spain	179 (5.14%)	Weill Cornell Med Coll	58 (1.67%)
9	Canada	172 (4.94%)	Massachusetts Gen Hosp	52 (1.49%)
10	Switzerland	152 (4.37%)	Univ Calif San Francisco	50 (1.44%)
Clinical field				
Rank	Country	N (%)	Institution	N (%)
1	United States	636 (53.13%)	Mem Sloan Kettering Canc Ctr	69 (5.76%)
2	Italy	135 (11.28%)	Dana Farber Canc Inst	68 (5.68%)
3	China	150 (12.53%)	Univ Texas MD Anderson Canc Ctr	53 (4.43%)
4	Germany	118 (9.86%)	Genentech Inc.	42 (3.51%)
5	France	116 (9.69%)	Harvard Med Sch	38 (3.17%)
6	Spain	93 (7.77%)	Mayo Clin	37 (3.09%)
7	United Kingdom	87 (7.27%)	NCI	37 (3.09%)
8	Belgium	83 (6.93%)	Univ Calif Los Angeles	30 (2.51%)
9	Canada	77 (6.43%)	Univ Libre Bruxelles	29 (2.42%)
10	Japan	74 (6.18%)	Sarah Cannon Res Inst	28 (2.34%)

**FIGURE 3:**
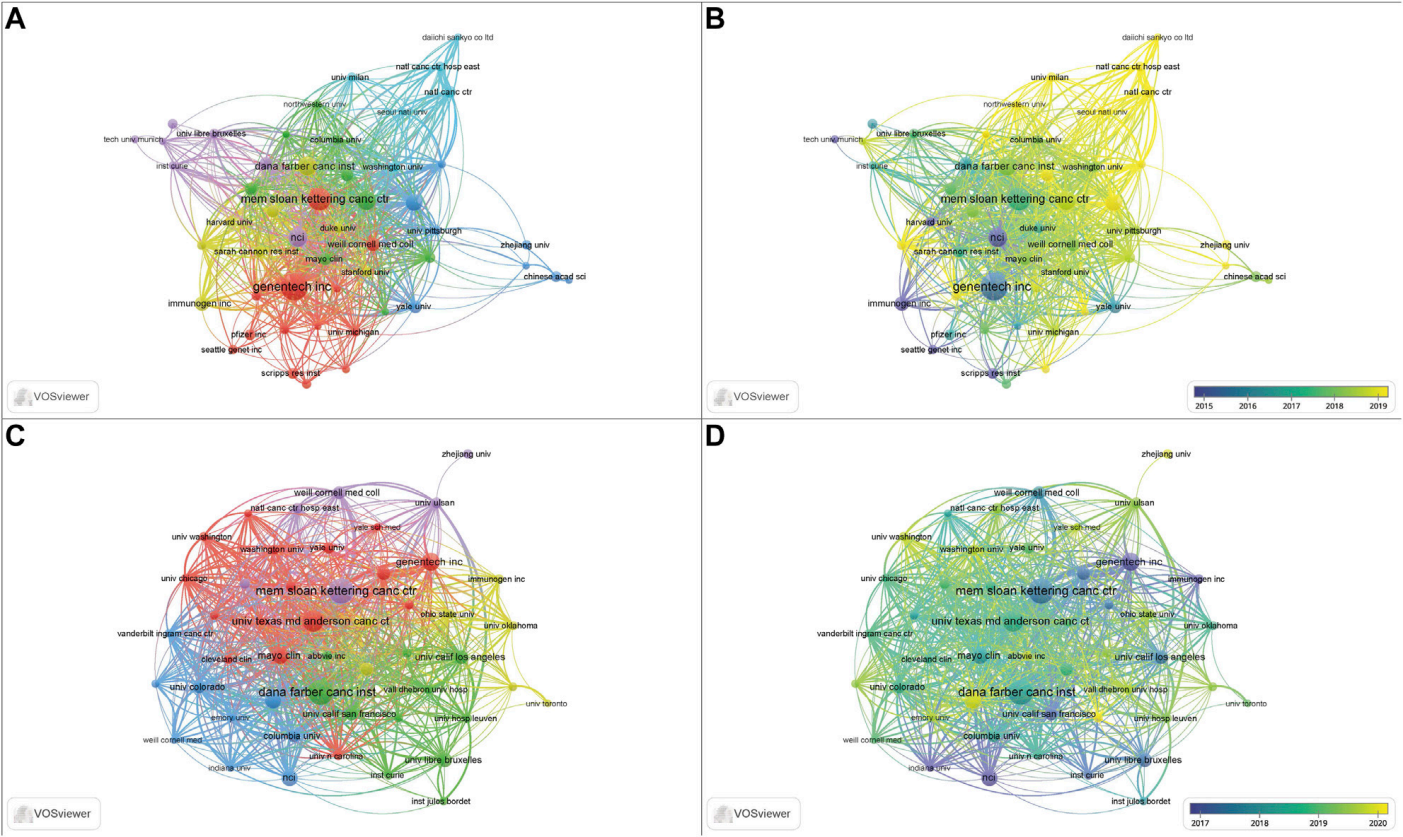
The collaboration and overlay map of institutions for ADC research in the solid cancer field. **(A,B)** The collaboration and overlay map of institutions for holistic ADC research, respectively. **(C,D)** The collaboration and overlay map of institutions for clinical ADC research, respectively.

A total of 4,410 institutions contributed to the 3,482 papers. The top 10 institutions contributed 897 (25.76%) papers, and these institutions are all located in the United States (Table 1). Among the top 10 institutions, Genentech Inc. (169, 4.85%) published the highest number of papers, followed by Mem Sloan Kettering Canc Ctr (130, 3.73%), Dana Farber Canc Inst (98, 2.81%), Univ Texas MD Anderson Canc Ctr (98, 2.81%), NCI (97, 2.79%), and Harvard Med Sch (82, 2.35%). Mem Sloan Kettering Canc Ctr (69, 5.76%) has published the most clinical studies, followed by Dana Farber Canc Inst (68, 5.68%), Univ Texas MD Anderson Canc Ctr (53, 4.43%), and Genentech Inc., (42, 3.51%). Figure 3 presents the cooperation ship between institutions and indicates that Mem Sloan Kettering Canc Ctr, Dana Farber Canc Inst, and Univ Texas MD Anderson Canc Ctr are the dominating centers for launching clinical trials of ADC drugs. The overlay map (Figure 3B,D) suggests that Univ Texas MD Anderson Canc Ctr starting later but growing rapidly in the ADC research field compared to the other three institutions.

### 3.4 Authors and Cited Authors

A total of 18,061 authors were obtained in the 3,482 publications. Table 2 shows the top 10 productive authors and the most cited authors. The top 10 authors contributed 239 papers. Krop IE (27 papers) published the highest number of papers, followed by Goldenberg DM and Zeglis BM (26 papers), Girish S, Lewis JS (25 papers), and Saint AD (23 papers). Among the top 10 cited authors, Krop, IE ranked first, with 2,333 local citations, followed by Guardino E (1415 local citations), Dieras V (1,267 local citations), and Sliwkowski MX (1,212 local citations), while the remaining authors had less than 1,200 local citations. As for clinical research, the most productive author was Goldenberg DM (19 papers), while the most cited author was Krop IE (696 local citations) followed by Kim SB (392 local citations) and Modi S (367 local citations).

**TABLE 2:** The top 10 authors and cited authors of ADC research in the solid cancer field [n (%)].

Holistic Field				Clinical Field			
Author	Record	Author	TLCS	Author	Record	Author	TLCS
Krop IE	27	Krop IE	2,333	Goldenberg DM	19	Krop IE	696
Goldenberg DM	26	Guardino E	1,415	Tolaney SM	17	Kim SB	392
Zeglis BM	26	Dieras V	1,267	Krop IE	15	Modi S	367
Girish S	25	Sliwkowski MX	1,212	Cortes J	14	Girish S	323
Lewis JS	25	Girish S	1,072	Harbeck N	14	Wildiers H	317
Santin AD	23	Welslau M	1,065	Hurvitz SA	14	Goldenberg DM	306
Beck A	22	Miles D	1,064	Sharkey RM	14	Hurvitz SA	293
Kozak KR	22	Gianni L	1,035	Bardia A	13	LoRusso PM	290
Tolaney SM	22	Blackwell K	1,008	Curigliano G	13	Sharkey RM	286
Cortes J/Curigliano G/Hurvitz SA	21	Baselga J	994	Santin AD	13	Guardino E	264
Sharkey RM/Senter PD	21	-	-	Shitara K	13	-	-

### 3.5 Journals and Cited Journals

**FIGURE 4:**
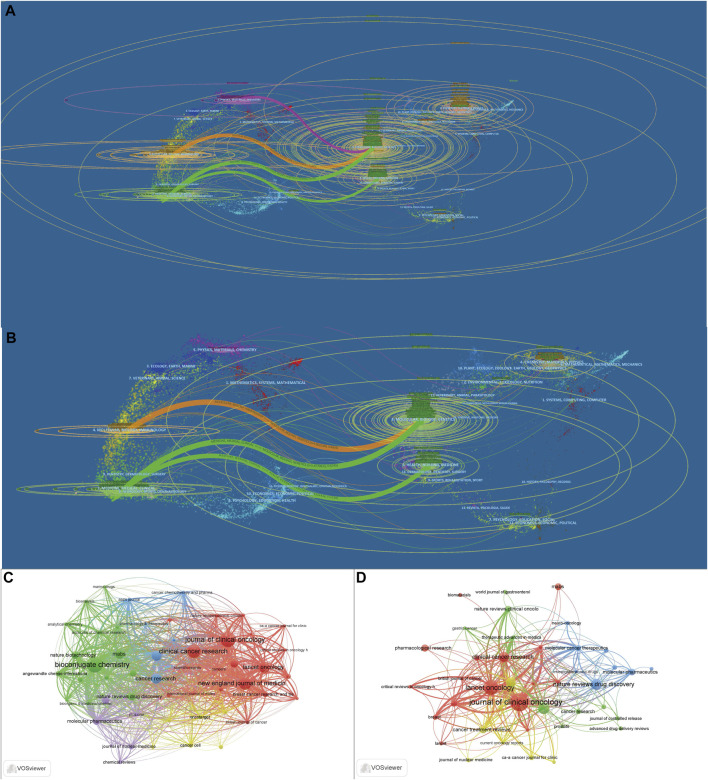
The dual-map overlay of journals and network map of cited journals related to ADC research in the solid cancer field. **(A,B)**. The dual-map overlay of journals for holistic and clinical research, respectively. **(C,D)**. The network map of cited journals for holistic and clinical research, respectively.

The 3,482 papers were published in 720 journals. Figure 4A,B presents the dual-map overlay of journals. The left side represents the map of citing journals and the right side represents the map of the cited journals. The label represents the subject covered by the journal. Colored curves represent paths of references, where each curve originates from the citing map and points to the cited map. There were four main citation paths in the holistic field and three main paths in the clinical field. Table 3 presents the top 10 journals and most cited journals. The top 10 journals contributed 658 (18.90%) papers. *Bioconjugate Chemistry* (114 papers) ranked first, followed by *Molecular Cancer Therapeutics* (98 papers), *Clinical Cancer Research* (90 papers), and *Cancers* (70 papers). As for clinical research, the top three productive journals are *Cancers* (33 papers), *Journal of Clinical Oncology* (30 papers), and *Clinical Cancer Research* (28 papers). The *Journal of Clinical Oncology* is the most-cited journal both in the holistic field (1787 local citations) and clinical field (817 local citations) of ADC research. *Bioconjugate Chemistry* (1,376 local citations) is the second cited journal in the holistic field, followed by *Clinical Cancer Research* (1,311 local citations) and *New England Journal of Medicine* (1,156 local citations). *Lancet Oncology* (585 local citations) is the second most cited journal in the clinical field, followed by *Clinical Cancer Research* (268 local citations), *New England Journal of Medicine* (204 local citations), and *Annals of Oncology* (158 local citations). Figure 4C,D shows the citation relationships between the journals.

**TABLE 3:** The top 10 journals and cited journals of ADC research in the solid cancer field.

Holistic Field					
Journal	IF	Record	Journal	IF	TLCS
BIOCONJUGATE CHEMISTRY	4.774	114	JOURNAL OF CLINICAL ONCOLOGY	44.544	1787
MOLECULAR CANCER THERAPEUTICS	6.261	98	BIOCONJUGATE CHEMISTRY	4.774	1,376
CLINICAL CANCER RESEARCH	12.531	90	CLINICAL CANCER RESEARCH	12.531	1,311
CANCERS	6.639	70	NEW ENGLAND JOURNAL OF MEDICINE	91.253	1,156
MOLECULAR PHARMACEUTICS	4.939	58	LANCET ONCOLOGY	41.316	1,042
MABS	5.857	54	MOLECULAR CANCER THERAPEUTICS	6.261	1,036
BREAST CANCER RESEARCH AND TREATMENT	4.872	44	CANCER RESEARCH	12.701	654
SCIENTIFIC REPORTS	4.38	44	NATURE BIOTECHNOLOGY	54.908	555
CANCER RESEARCH	12.701	43	MOLECULAR PHARMACEUTICS	4.939	386
JOURNAL OF CLINICAL ONCOLOGY	44.544	43	NATURE REVIEWS DRUG DISCOVERY	84.694	385
Clinical field					
Journal	IF	Record	Journal	IF	TLCS
CANCERS	6.639	33	JOURNAL OF CLINICAL ONCOLOGY	44.544	817
JOURNAL OF CLINICAL ONCOLOGY	44.544	30	LANCET ONCOLOGY	41.316	585
CLINICAL CANCER RESEARCH	12.531	28	CLINICAL CANCER RESEARCH	12.531	268
LANCET ONCOLOGY	41.316	26	NEW ENGLAND JOURNAL OF MEDICINE	91.253	204
EXPERT OPINION ON BIOLOGICAL THERAPY	4.388	23	ANNALS OF ONCOLOGY	32.976	158
BREAST CANCER RESEARCH AND TREATMENT	4.872	22	BREAST CANCER RESEARCH AND TREATMENT	4.872	154
MOLECULAR CANCER THERAPEUTICS	6.261	21	NATURE REVIEWS DRUG DISCOVERY	84.694	81
FRONTIERS IN ONCOLOGY	6.244	20	MOLECULAR CANCER THERAPEUTICS	6.261	80
THERAPEUTIC ADVANCES IN MEDICAL ONCOLOGY	8.168	19	CANCER	6.86	72
ANNALS OF ONCOLOGY	32.976	18	JOURNAL OF MEDICINAL CHEMISTRY	7.446	42
FUTURE ONCOLOGY	3.404	18	-	-	-

### 3.6 Cited References and References With Citation Bursts

Table 4 presents the top 10 cited references from 2011 to 2022. These references suggest that T-DM1 for HER2-positive breast cancer is a focus of interest for researchers. There are eight clinical trials associated with the therapeutic effects evaluation of trastuzumab emtansine in HER2-positive breast cancer patients (Burris et al., 2011; Krop et al., 2012; Verma et al., 2012; Hurvitz et al., 2013; Krop et al., 2014; Diéras et al., 2017; Krop et al., 2017; Perez et al., 2017), and the most cited one was a phase 3 clinical trial, which demonstrated that T-DM1 prolonged progression-free survival of breast cancer patients who had been previously treated with trastuzumab and a taxane (Verma et al., 2012). Junttila et al. (2011) and Lorusso et al. (2011) reviewed the mechanisms and clinical progress of T-DM1, respectively. Trastuzumab deruxtecan is another trastuzumab-based ADC agent. Ogitani et al. (2016) evaluated its pharmacologic activities with HER2-positive cell lines and patient-derived xenograft models, while Modi et al. (2020) demonstrated its durable antitumor activity in HER2-positive metastatic breast cancer patients in the DESTINY-Breast01 trial. The second most cited paper was written by Beck et al. (2017), who reviewed the progresses of first- and second-generation ADCs as well as envisaged third-generation ADCs. Shen et al. (2012) assessed the impact of the conjugation site on ADCs.

**TABLE 4:** The top 10 cited references related to ADC research in the solid cancer field from 2011–2022.

Holistic Field		
Rank	Author/Year/Journal/Volume/Page/DOI	TLCS
1	Verma S, 2012, NEW ENGL J MED, V367, P1783, DOI 10.1056/NEJMoa1209124	765
2	Beck A, 2017, NAT REV DRUG DISCOV, V16, P315, DOI 10.1038/nrd.2016.268	295
3	Krop IE, 2014, LANCET ONCOL, V15, P689, DOI 10.1016/S1470-2045 (14)70,178–0	238
4	Burris HA, 2011, J CLIN ONCOL, V29, P398, DOI 10.1200/JCO. 2010.29.5865	202
5	Shen BQ, 2012, NAT BIOTECHNOL, V30, P184, DOI 10.1038/nbt.2108	187
6	Modi S, 2020, NEW ENGL J MED, V382, P610, DOI 10.1056/NEJMoa1914510	186
7	Junttila TT, 2011, BREAST CANCER RES TR, V128, P347, DOI 10.1007/s10549-010-1090-x	180
8	LoRusso PM, 2011, CLIN CANCER RES, V17, P6437, DOI 10.1158/1,078–0,432.CCR-11–0,762	158
9	Ogitani Y, 2016, CLIN CANCER RES, V22, P5097, DOI 10.1158/1,078–0,432.CCR-15–2,822	153
10	Dieras V, 2017, LANCET ONCOL, V18, P732, DOI 10.1016/S1470-2045 (17)30,312–1	145
Clinical field		
Rank	Author/Year/Journal/Volume/Page/DOI	TLCS
1	Verma S, 2012, NEW ENGL J MED, V367, P1783, DOI 10.1056/NEJMoa1209124	372
2	Krop IE, 2014, LANCET ONCOL, V15, P689, DOI 10.1016/S1470-2045 (14)70,178–0	143
3	Modi S, 2020, NEW ENGL J MED, V382, P610, DOI 10.1056/NEJMoa1914510	99
4	Burris HA, 2011, J CLIN ONCOL, V29, P398, DOI 10.1200/JCO. 2010.29.5865	95
5	Dieras V, 2017, LANCET ONCOL, V18, P732, DOI 10.1016/S1470-2045 (17)30,312–1	90
6	Krop IE, 2017, LANCET ONCOL, V18, P743, DOI 10.1016/S1470-2045 (17)30,313–3	85
7	Junttila TT, 2011, BREAST CANCER RES TR, V128, P347, DOI 10.1007/s10549-010-1090-x	78
8	Hurvitz SA, 2013, J CLIN ONCOL, V31, P1157, DOI 10.1200/JCO. 2012.44.9694	77
9	Perez EA, 2017, J CLIN ONCOL, V35, P141, DOI 10.1200/JCO. 2016.67.4887	73
10	Krop IE, 2012, J CLIN ONCOL, V30, P3234, DOI 10.1200/JCO. 2011.40.5902	71

**FIGURE 5:**
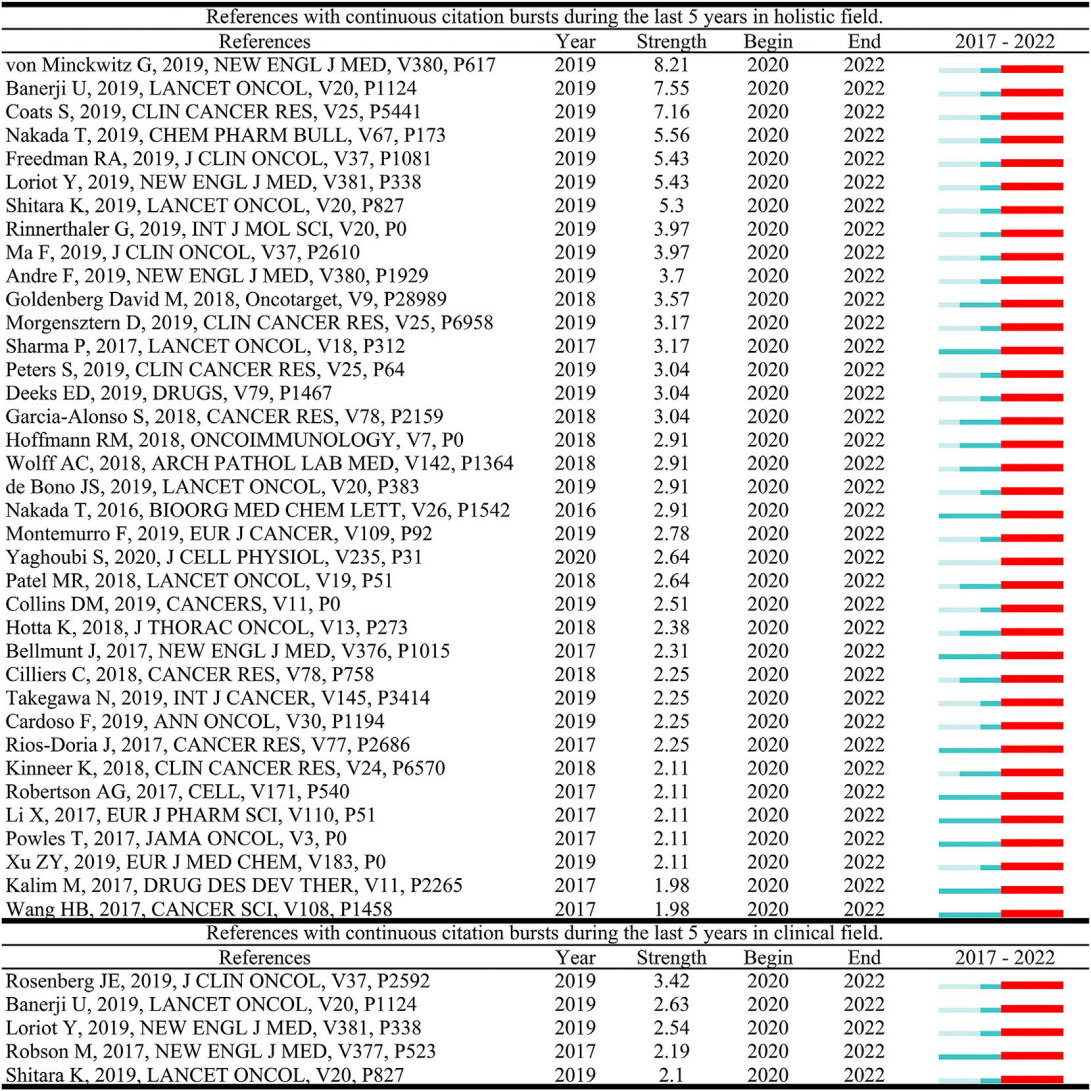
References with continuous citation bursts during the last 5 years.

References with citation bursts are defined as those that are cited frequently over a while. In CiteSpace, the timespan was set as 2017–2022 and references with a burst termination date of 2022 remained. In Figure 5, the blue line represents the time interval. The time in which a reference was found to have a burst is displayed by a red line, indicating the first year and the last year of the duration of the burst. A total of 37 burst references and five burst references were detected in the holistic field and clinical field of ADC research, respectively. The reference with the strongest citation burst documented the efficacy of T-DM1 in the treatment of residual invasive HER2-positive breast cancer (Von Minckwitz et al., 2019), while in the clinical field, Rosenberg et al. (2019) reported the efficacy and safety data of enfortumab vedotin, an ADC drug that targets nectin-4, in the treatment of previously chemotherapy and immunotherapy treated urothelial carcinoma.

### 3.7 Co-occurrence Keywords and Cluster Analysis

**FIGURE 6:**
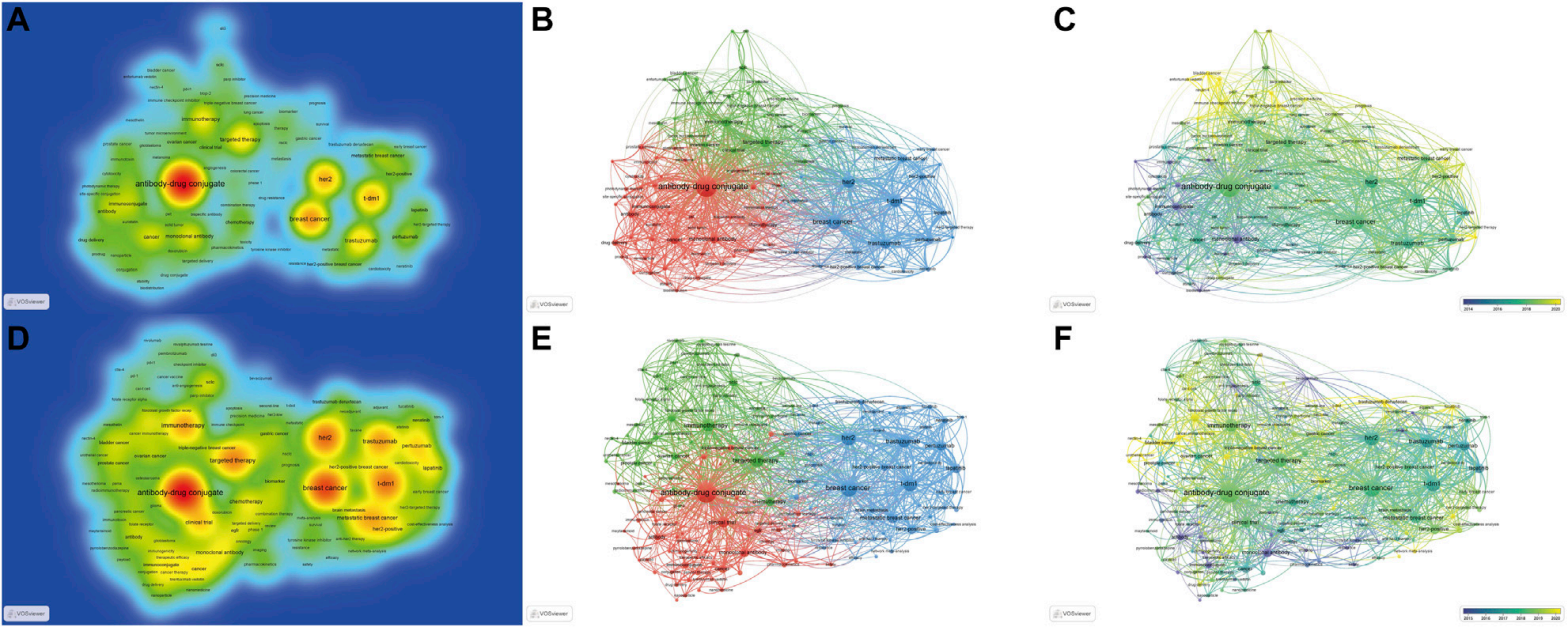
The density, co-occurrence, and overlay map of keywords. (The density, co-occurrence, and overlay map are displayed in the first to third columns, respectively.) **(A–C)**. Maps for the holistic ADC research field. **(D–F)**. Maps for the clinical ADC research field.

A total of 4,634 keywords were extracted from the 3,482 papers. Figure 6 shows the density map, co-occurrence map, and overlay map for the keyword. Table 5 presents the top 20 occurring keywords. Keywords with high occurrence in the clinical field were consistent with the holistic field. Antibody-drug conjugate, breast cancer, HER2, T-DM1, metastatic breast cancer, ovarian cancer, and small cell lung cancer were greatly concerned. As can be seen from Figure 6, the ADC research field has been extended to triple-negative breast cancer, prostate cancer, gastric cancer, bladder cancer, urothelial carcinoma, pancreatic cancer, colorectal cancer, melanoma, glioblastoma, and non-small-cell lung cancer. Multifarious ADC agents including T-DM1, trastuzumab deruxtecan, sacituzumab govitecan, enfortumab vedotin, and rovalpituzumab tesirine were observed. Several targets of ADCs are of eminent interest, such as HER2, trophoblast cell-surface antigen (trop2), delta-like ligand 3 (dll3), and nectin-4.

**TABLE 5:** The top 20 keywords in terms of the occurrence of ADC research in the solid cancer field.

Holistic Field		Clinical Field	
Label	Occurrences	Label	Occurrences
antibody-drug conjugate	756	antibody-drug conjugate	235
breast cancer	332	breast cancer	172
her2	288	her2	132
t-dm1	270	t-dm1	124
trastuzumab	212	targeted therapy	109
targeted therapy	196	trastuzumab	96
immunotherapy	163	immunotherapy	91
monoclonal antibody	139	clinical trial	70
cancer	133	metastatic breast cancer	69
metastatic breast cancer	106	pertuzumab	60
pertuzumab	106	her2-positive	52
immunoconjugate	98	lapatinib	48
antibody	91	chemotherapy	44
chemotherapy	80	monoclonal antibody	44
her2-positive	78	cancer	39
lapatinib	74	her2-positive breast cancer	36
clinical trial	71	immune checkpoint inhibitor	36
ovarian cancer	60	sclc	35
sclc	58	ovarian cancer	28
drug delivery	53	antibody	26

A clustering analysis was performed based on the co-occurrence of keywords and a total of 3 clusters were identified. As we can see from Figure 6B, the red cluster is the largest cluster, which contains 49 keywords, mainly including antibody-drug conjugate, immunoconjugate, monoclonal antibody, drug delivery, pharmacokinetics, prostate cancer, linker, and targeted delivery. The blue cluster includes 25 keywords, which primarily consist of breast cancer, HER2, T-DM1, metastatic breast cancer, gastric cancer, and trastuzumab deruxtecan. The green cluster is composed of targeted therapy, immunotherapy, clinical trial, ovarian cancer, small cell lung cancer, triple-negative breast cancer, bladder cancer, trop2, urothelial carcinoma, sacituzumab govitecan, non-small cell lung cancer, apoptosis, dll3, enfortumab vedotin, and biomarker et al.

An overlay map can present the dynamic development process of a given research field. The terms Immunoconjugate, conjunction, and monoclonal antibody indicate the focus of original research on ADC. Antibody-drug conjugate, HER2, breast cancer, T-DM1, target therapy, ovarian cancer, and small cell lung cancer are at intermediate, while gastric cancer, triple-negative breast cancer, bladder cancer, urothelial carcinoma, nectin-4, dll3, sacituzumab govitecan, and trastuzumab deruxtecan are novel topics.

The knowledge map for the clinical research field provides a similar keyword distribution to the holistic field, while we can obtain a more specific perspective of clinical research of ADC in the field of solid cancer from Figure 6D–F.

### 3.8 The Top Five Targets, Payloads and Linkers of ADC

The composition of the ADC structure was retrieved on WoSCC and the top five targets, payloads, and linkers, including their publications and citations, are presented in Table 6. HER2 is the most investigated target with 883 publications and 29,924 citations in the ADC field, followed by trop2 (93 papers and 2,239 citations) and mesothelin (62 papers and 1,632 citations). The most studied payload is maytansinoid with 1,009 papers published and 41,928 citations, followed by auristatin (596 papers and 25,027 citations), and calicheamicin (197 papers and 15,088 citations). Peptide linker (467 papers and 13,279 citations) has the highest publications and citations, followed by disulfide linker (231 papers and 10,938 citations), and thioether linker (50 papers and 3,047 citations).

**TABLE 6:** The top five most investigated targets, payloads, and linkers of ADC.

Target	Record	Citations	Payload	Record	Citations	Linker	Record	Citations
HER2	883	29,924	Maytansinoid	1,009	41,928	Peptide	467	13,279
TROP2	93	2,239	Auristatin	596	25,027	Disulfide	231	10,938
Mesothelin	62	1,632	Calicheamicin	197	15,088	Thioether	50	3,047
DLL3	49	1776	Pyrrolobenzodiazepine	186	4,652	Hydrazone	30	2,143
Nectin-4	47	730	Camptothecin	89	3,118	β-glucuronide	21	520

## 4 Discussion

In the current study, we conducted a bibliometric analysis to reveal the research trend and frontier topic of ADC research in the field of solid cancer for the first time. A pivotal step forward toward ADC was the invention of monoclonal antibodies, which can bind antigenic epitopes specifically (Köhler and Milstein, 1975). The concept of “magic bullet,” which means that chemicals specifically and efficiently target tumor cells, contributes to the emergence of ADC (Schwartz, 2004). There are currently three generations of ADC drugs. In the first generation of ADC drugs, antitumor agents such as mitomycin C, idarubicin, anthracyclines, N-acetylmaran, adriamycin, perillyl alkaloids, and methotrexate are conjugated to murine monoclonal antibodies or humanized monoclonal antibodies mainly through non-cleavable linkers (Ponziani et al., 2020). The conjugating approach is random, resulting in uncontrollable drug-to-antibody ratio (DAR) values, which influence the release of the payload (Deslignière et al., 2021). Gemtuzumab ozogamicin was a first-generation ADC drug, which was approved for acute myeloid leukemia in 2000 (Norsworthy et al., 2018). However, it was withdrawn by Pfizer in 2010 due to its limited efficacy and redundant toxicity (Beck et al., 2010). The second-generation of ADCs possess increased cytotoxic drug conjugation levels and more-stable linkers compared to the first-generation and have shown significant clinical efficacy and safety performance (Beck et al., 2017). For the second generation of ADC drugs, humanized monoclonal antibodies are prudently selected, payloads with enhancive toxicity such as microtubule protein inhibitors are introduced, and reduced hinge cysteine conjugation is engineered, which largely improves the efficacy, safety, and chemistry, manufacturing and controls properties (Donaghy, 2016). However, the DAR values range from 0 to 8 (Lyon et al., 2015). Brentuximab vedotin and T-DM1 both belong to the second-generation ADC drug, and they have been approved for lymphoma and breast cancer by the FDA (Nagayama et al., 2017). The third generation ADC drug ameliorates the shortcomings of the previous generations. The site-specific binding of small molecule drugs to monoclonal antibodies is designed to produce third-generation ADCs, bringing about DARs of 2 or 4 (Martin et al., 2018). This generation of ADC drugs has improved stability, pharmacokinetics, and anti-tumor activity (Beck et al., 2017). Based on previous knowledge, lessons learned are now being concentrated on the development of third-generation ADCs, and numerous ADCs for solid tumors are under investigation. Our results suggested that there was a significantly increasing number of publications in the field, especially from 2018.

The United States was the most productive country and dominated the countries’ collaboration. Genentech Inc., and Mem Sloan Kettering Canc Ctr were the most productive institutions in the holistic field and clinical field of the ADC research, respectively, and both of them are located in the United States. Genentech Inc.’s reputation in the ADC field is ascribed to the design of T-DM1, which has been approved for advanced and early breast cancer in 2013 and 2019 by the FDA (Boyraz et al., 2013; Von Minckwitz et al., 2019). Aside from that, they’ve studied T-DM1 as an adjuvant for breast cancer, as well as its cost-effectiveness and drug resistance in recent years (Boyer et al., 2021; Mamounas et al., 2021; Sussell et al., 2021). Genentech Inc., also devotes itself to studying the mechanisms, pharmacokinetics, and structures of ADCs and to developing novel ADC agents. The phase I dose-escalation study of DMUC4064A, an innovative ADC drug that targets MUC16 expressed in the ovarian cancer cell, was completed in 2021 (Liu et al., 2021). Mem Sloan Kettering Canc Ctr released the results of its first ADC clinical trial in 2008, the phase I trial of MLN2704, a prostate-specific membrane antigen-targeted immunoconjugate, in the treatment of prostate cancer (Galsky et al., 2008). Over the past few years, Mem Sloan Kettering Canc Ctr conducted clinical trials in various solid cancers, including epithelial cancer, ovarian cancer, urothelial carcinoma, and early-stage HER2-positive breast cancer. The results of the IMMU-132-01 trial, FORWARD I trial, EV-201 trial, and ATEMPT trial were disclosed in 2021 (Bardia et al., 2021; Moore et al., 2021; Ruddy et al., 2021; Yu et al., 2021). Furthermore, the most cited paper published by Mem Sloan Kettering Canc Ctr was the clinical reports of the DESTINY-Breast01 trial (Modi et al., 2020).

Krop IE was the most productive and cited author in the holistic field, who has participated in several crucial clinical trials for T-DM1 in the treatment of HER2-positive breast cancer, such as the EMILIA (Verma et al., 2012) and TH3RESA (Krop et al., 2014) trials. Besides T-DM1, he has recently been involved in preliminary clinical trials of other HER2-targeted ADC agents, such as trastuzumab deruxtecan and MM-302. (Munster et al., 2018; Tamura et al., 2019). His latest research assessed the impact of HER2 heterogeneity in patients who had received T-DM1 and pertuzumab therapy, and the results indicated that HER2 heterogeneity was associated with resistance to HER2-targeted therapy (Filho et al., 2021). Goldenberg DM was the most productive author in the clinical field. He has been focusing on the development and clinical research of ADC drugs targeting trop2, whose initial study assessed the anti-tumor efficacy of an SN-38-anti-trop2 ADC in human cancer xenograft models and monkeys (Cardillo et al., 2011). In recent years, a mature anti-trop2 ADC drug named sacituzumab govitecan has been applied in the clinical research of triple-negative breast cancer (Bardia et al., 2019), NSCLC (Heist et al., 2017) and SCLC (Gray et al., 2017) by Goldenberg DM, as well as another anti-trop2 ADC drug named labetuzumab govitecan in colorectal cancer (Dotan et al., 2017).

*Bioconjugate Chemistry*, a journal aiming to present the preparation, properties, and applications of biomolecular conjugates, has the most papers published. The paper with the topmost citations in the past decade published by *Bioconjugate Chemistry* introduced a cell-free protein expression system designed for the rapid synthesis of ADCs through site-specific incorporation of the para-azidomethyl-L-phenylalanine (Zimmerman et al., 2014). The second most cited paper measured the surface expression of trop2 in a wide range of human solid cancers and the pharmacokinetics of sacituzumab govitecan (Cardillo et al., 2015). The *Journal of Clinical Oncology* was the most cited journal, which published its first clinical paper about ADC drugs in 1999 and kept presenting substantial clinical research. The results of the MARIANNE study were reported in 2017 in the *Journal of Clinical Oncology*, which immediately elicited eminent concerns (Perez et al., 2017).

References and keywords analysis reflect current and future research topics jointly. The monoclonal antibody in the ADC structure generally determines the range of applicability of an ADC. A total of five targets, including HER2, trop2, mesothelin, nectin-4, and dll3 were presented as high-occurrence keywords in our knowledge map. HER2 is a pivotal target for ADC drugs. The alteration of HER2 occurs in a wide range of solid cancers and it can cause increased downstream signaling, which further results in cell growth, metastasis, drug resistance, and angiogenesis (Zhao and Xia, 2020). Breast cancers are the main battleground for HER2-targeted ADC drugs, consequentially. There are already two ADC drugs, T-DM1 and trastuzumab deruxtecan, approved for HER2-positive breast cancers by FDA. However, breast cancer patients with low or negative HER2 expression remain challenging. The next generation of ADCs have the potential to be active in these populations due to their advanced pharmaceutical properties, such as trastuzumab duocarmazine, which showed notable clinical activity in HER2-low expression breast cancer patients in a phase 1 trial (Banerji et al., 2019; Ferraro et al., 2021). In addition, gastrointestinal cancer, NSCLC, endometrial cancer, and urothelial carcinoma also express HER2 on the cell surface, and early clinical trials have been conducted with HER2-targeted ADC drugs (Tsurutani et al., 2020). ADCs that target antigenic epitopes beyond HER2 have opened up a slew of new clinical possibilities for triple-negative breast cancer. Trop2 is a transmembrane glycoprotein that is overexpressed in a variety of solid cancers such as breast cancer, lung cancer, and urothelial carcinoma with minimal or no baseline expression in normal tissues. Previous studies have proved that trop2 is associated with tumor progression and the development of metastases (Ripani et al., 1998; Bignotti et al., 2012; Trerotola et al., 2013). Trop2-targeted ADC drugs mainly include sacituzumab govitecan, PF-06664178, and datopotamab deruxtecan, of which sacituzumab govitecan and datopotamab deruxtecan have presented appealing clinical efficacy in solid cancers, while PF-06664178 has failed in a phase I trial (Liao et al., 2021). The response rate of heavily pretreated metastatic triple-negative breast cancer patients who received sacituzumab govitecan-hziy was 33.3%, and the median overall survival was 13.0 months. Sacituzumab govitecan-hziy prolonged the duration of treatment when compared with the immediate previous antitumor therapy (5.1 vs 2.5 months) (Bardia et al., 2019). As a result, sacituzumab govitecan-hziy was granted accelerated approval for triple-negative breast cancer by the FDA in 2020. Mesothelin is a 40-kDa cell surface glycoprotein, which is highly expressed in several human cancers (Chang and Pastan, 1996), including lung cancer (∼50% of cases) (Ordóñez, 2003), ovarian cancer (∼70% of cases) (Hassan et al., 2005) and pancreatic/biliary adenocarcinomas (∼100% of cases) (Argani et al., 2001). Two mesothelin-targeted ADC drugs including Anetumab ravtansine and DMOT4039A have presented active anti-tumor effects in the preclinical studies and further clinical trials are in progress (Singh et al., 2021). Nectin-4 is a member of the nectin family and it is weakly expressed in normal tissues while highly expressed in various tumor cells, including urothelial, lung, breast, and ovarian cancers. Urothelial carcinoma patients with overexpressed nectin-4 had a significantly worse prognosis. Nectin-4-targeted ADC therapy was applied in urothelial carcinoma. Enfortumab vedotin was approved for heavily pretreated locally advanced or metastatic urothelial cancer patients by the FDA in 2019 (Moussa et al., 2021), and it is currently being tested for solid cancers in a phase II study (Bruce et al., 2020). DLL3 is an inhibitory notch ligand that is highly expressed in SCLC but minimally expressed in normal lung tissues (Saunders et al., 2015). Overexpression of DLL3 was associated with irrepressible migration and invasion of SCLC (Furuta et al., 2019). Therefore, DLL3 was regarded as an emerging target for SCLC. Rovalpituzumab tesirine is a DLL3-targeted ADC. Although rovalpituzumab tesirine represented satisfactory anti-tumor effects in preclinical models (Saunders et al., 2015), its phase 3 trial was halted due to shorter overall survival when compared with topotecan (Owen et al., 2019). Researchers are testing rovalpituzumab tesirine in different disease settings of SCLC, and a clinical study of rovalpituzumab tesirine as maintenance therapy for SCLC is ongoing (Owen et al., 2019). Tissue factor has been a promising topic in recent years, and one of our burst references detailed a clinical trial about tissue factor-targeted ADC drugs. The tissue factor is a transmembrane glycoprotein that functions not only as the main initiator of extrinsic coagulation pathways but also as a promoter of tumor progression. Tissue factor is overexpressed in a variety of solid cancers, such as cervical cancer (Cocco et al., 2011), NSCLC (Koomägi and Volm, 1998), endometrial cancer (Fadare et al., 2011), prostate cancer (Akashi et al., 2003) and ovarian cancer (Abu Saadeh et al., 2013). The phase 1–2 trial of tisotumab vedotin, a tissue factor targeted-ADC drug, indicated that tisotumab vedotin had encouraging preliminary antitumor activity in solid cancers with an objective response of 15.6% (De Bono et al., 2019).

Burst references indicated several future research concerns. Acquired resistance is a future challenge for ADC therapy. Garcia-Alonso et al. (2018) reviewed the mechanisms of resistance to ADCs and raised strategies to overcome resistance. Collins et al. (2019) introduced the progress of combination therapy, which is regarded as a potential approach to avoiding acquired resistance. Previous *in vivo* studies demonstrated that ADCs synergized with PD-1 antibodies to exert antitumor effects, which supported a combination treatment strategy of ADC therapy and PD-1 inhibitors (Rios-Doria et al., 2017). Kinneer et al. (2018) and Wang et al. (2017) discovered that the expression of SLC46A3 and the activity of V-ATPase in lysosomes could be used as biomarkers for predicting T-DM1 resistance, respectively. Studies were also conducted to identify biomarkers that can recognize the beneficiary population of ADC therapies. Takegawa et al. (2019) reported that colorectal cancer patients who expressed HER2 protein without HER2 amplification might be sensitive to trastuzumab deruxtecan. The accumulation of knowledge on the structure, mechanism, and pharmacokinetics of ADCs has facilitated the development of the next generation of ADCs. Yaghoubi et al. (2020) and Hoffmann et al. (2018) summarized the cytotoxic small molecule drugs that were potentially processed into payloads, and the characteristics of engineering antibodies in ADCs, respectively. Nakada et al. (2016) reviewed the progress of ADCs that contained exatecan derivative-based cytotoxic payloads. In addition, Kalim et al. (2017) illustrated endocytosis and intracellular trafficking of ADCs, which was the approach by that ADCs entered tumor cells, while Cilliers et al. (2018) demonstrated that the intratumoral distribution of ADCs plays a major role in ADC efficacy. Both of them have enlightened the future development of ADCs.

## 5 Conclusion

In this study, we conducted a bibliometric analysis to reveal the research trend of ADCs in the field of solid cancer. Publications associated with this field are increasing rapidly. Further clinical trials of ADCs and designs of the next-generation of ADCs are the current focuses of the field. Acquired resistance to ADCs and biomarkers for ADC therapy efficacy monitoring are future concerns.

## Data Availability

The original contributions presented in the study are included in the article/Supplementary Material, further inquiries can be directed to the corresponding author.
