# Supplementary material for: Research Trend of Publications Concerning Antibody-Drug Conjugate in Solid Cancer: A Bibliometric Study
Source: Front Pharmacol. 2022 Jun 20;13:921385. doi: 10.3389/fphar.2022.921385 (PMC9252465; doi:10.3389/fphar.2022.921385)
Supplement: Supplementary file 1 [file Table1.DOCX]

Supplementary Table 1. Retrieval strategy and result.

| Step | N | Retrieval strategy |
| --- | --- | --- |
|  | 3691326 | TS = (*cancer* OR *neoplas* OR *tumo* OR *carcinoma* OR *adenocarcinoma* OR *metasta* OR *malignan* OR *sarcoma* OR *melanoma* OR *oncolog*) |
|  | 475762 | TS = (*hodgkin* OR *nonhodgkin* OR *leukemia* OR *lymphoma* OR *myeloma* OR hematologic malignan* OR hematologic neoplas*) |
|  | 3458727 | #1 NOT #2 |
|  | 9847 | TS = (“antibody* drug conjugate*” OR Immunoconjugate* OR Trastuzumab deruxtecan OR DS-8201a OR Enhertu OR T-Dxd OR Lifastuzumab vedotin OR LIFA OR DNIB0600A OR Anetumab ravtansine OR BAY 94–9343 OR PF-06664178 OR RN927C OR IMGN853 OR Mirvetuximab soravtansine OR MIRV OR ABT-414 OR Depatuxizumab mafodotin OR Depatux-M OR Telisotuzumab Vedotin OR ABBV399 OR Teliso-V OR PF-06647263 OR PF-06263507 OR Enfortumab vedotin OR Brentuximab vedotin OR ADCETRIS OR Aprutumab Ixadotin OR BAY1187982 OR Rovalpituzumab tesirine OR SC16LD6.5 OR Rova T OR Sacituzumab govitecan OR IMMU-132 OR HRS7-SN38 OR Trodelvy OR Lorvotuzumab mertansine OR IMGN901 OR Trastuzumab emtansine OR T-DM1 OR Kadcyla OR Ado-Trastuzumab emtansine OR TAK-264 OR MLN0264 OR IIndusatumab Vedotin OR 5F9vcMMAE OR LMB-100 OR Labetuzumab Govitecan OR IMMU-130 OR Tisotumab Vedotin OR Tivdak OR TF-011-MMAE OR AMG 172 OR AGS-16M8F OR AGS-16C3F OR SGN-CD70A OR PSMA ADC OR DSTP3086S OR ASG-5ME OR Glembatumumab vedotin OR CDX-011 OR CR011-vcMMAE OR AMG 595) |
|  | 3482 | #3 AND #4 (Document types: Articles or Review Articles) |
|  | 817195 | TS = (meta analysis OR systematic review OR metaanaly* OR overview* OR (pooled AND analys*)) |
|  | 1409485 | TS = trial* |
|  | 2039997 | #6 OR #7 |
|  | 1197 | #8 AND #5 |
